# Synergetic antibacterial activity of reduced graphene oxide and boron doped diamond anode in three dimensional electrochemical oxidation system

**DOI:** 10.1038/srep10388

**Published:** 2015-05-21

**Authors:** Xiujuan Qi, Ting Wang, Yujiao Long, Jinren Ni

**Affiliations:** 1 School of Environment and Energy, Peking University Shenzhen Graduate School, Shenzhen 518055, China; 2 College of Environmental Science and Engineering, Peking University; Key Laboratory of Water and Sediment Sciences, Ministry of Education, Beijing 100871, China

## Abstract

A 100% increment of antibacterial ability has been achieved due to significant synergic effects of boron-doped diamond (BDD) anode and reduced graphene oxide (rGO) coupled in a three dimensional electrochemical oxidation system. The rGO, greatly enhanced by BDD driven electric field, demonstrated strong antibacterial ability and even sustained its excellent performance during a reasonable period after complete power cut in the BDD-rGO system. Cell damage experiments and TEM observation confirmed much stronger membrane stress in the BDD-rGO system, due to the faster bacterial migration and charge transfer by the expanded electro field and current-carrying efficiency by quantum tunnel. Reciprocally the hydroxyl-radical production was eminently promoted with expanded area of electrodes and delayed recombination of the electron–hole pairs in presence of the rGO in the system. This implied a huge potential for practical disinfection with integration of the promising rGO and the advanced electrochemical oxidation systems.

High efficient disinfection has received extensive attention for drinking water security. Recently, electrochemical disinfection process has been reported to be convenient but highly efficient to produce germ-free water^1^. Compared with conventional disinfections technologies, such as chlorine dosing disinfection and ozone disinfection, electrochemical disinfection is a green technology without bringing additional chemical compounds to the water treated or producing hazardous byproduct. Boron-doped diamond (BDD) is a typical material to serve as anode in the electrochemical disinfection process, and is reported to be effective for bacterial inactivation^2^. Due to its wide potential window, corrosion stability, inert surface and strong oxidation capacity, BDD anode shows great advantages over other electrode materials^3^,^4^. In a chloride-free disinfection system, the electro-generated hydroxyl radical (•OH) is the major component^5^,^6^ to kill the bacterial. In contrast to Ti/RuO_2_, Ti/IrO_2_, Ti/Pt–IrO_2_, and Pt as anode materials, BDD produced the most amount of •OH in chloride-free electrolyte. However, BDD anode required larger driving potentials, thus increasing the energetic requirements. Previous studies demonstrated that carbon materials incorporated to the BDD system would significantly promote current efficiency due to expanding of electrolytic cell specific surface area and thus reduce the driving potentials and energy consumption^7^. Vecitis *et al.* has reported the synergic effect from multiwalled carbon nanotubes in the *E. coli* removal and inactivation^8^. Rahaman *et al.* proved that, viral removal is markedly increased in the electrochemical multiwalled carbon nanotube (EC-MWNT) system due to the electrostatic interactions with anodic MWNTs^9^.

As a well-studied carbon nanomaterial, graphene shows fantastic properties, such as the dramatic mechanical strength, perfect thermal stability and excellent electronic mobility. Remarkably, Fan’s group^10^ first found the cytotoxicity from graphene toward bacteria. The antimicrobial mechanism mainly included direct membrane contact with sharp nanosheets^11^ and charge transfer^12^,^13^ between graphene and membrane. The reduced graphene oxide (rGO) sheets are reported to have better cytotoxicity and show an inhibition to proliferation of the bacteria on their surfaces^14^. Tu *et al.*^15^ studied the underlying interaction between rGO and cell membranes experimentally and theoretically, which highlighted that graphene nanosheets can penetrate into the cells and extract large amounts of phospholipids from the cell membranes because of the strong dispersion interactions between graphene and lipid molecules.

Since the graphene (including rGO) displayed obvious cytotoxicity toward bacteria, herein we build a BDD-rGO three dimensional electrochemical oxidation system to explore the antibacterial ability under the effect of electric field with the presence of rGO. Synergetic antibacterial ability was studied by both the sustained antibacterial ability of rGO after complete power cut and reciprocal promotion of hydroxyl-radical production in BDD-rGO system. The underlying disinfection mechanism based on membrane stress due to lager bacterial migration and charge transfer was also discussed through cell damage experiments and TEM detection. The new sight of antibacterial mechanism by rGO under the electro field especially from the view of cells implied a better understanding of cell death in a synergy system with chemical free radicals oxidation and physical disruption.

## Results

### Morphology of rGO

**Figure 1:**
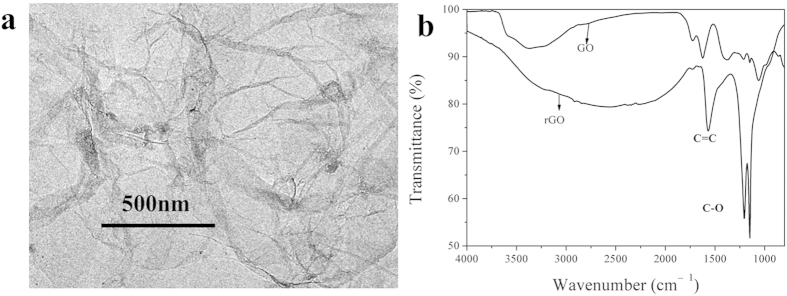
(**a**) TEM image of rGO; (**b**) FT-IR ATR of GO and rGO.

After the reduction by hydrazine, the brown GO dispersion turned into opaque and black rGO dispersion (Fig. S1). The rGO dispersion can be homogeneous for several days and not precipitated even under 6000 rpm centrifugation for 30 minutes. Figure 1a shows the TEM image of rGO nanosheets dried upon formvar grids. Small wrinkles can be seen, which indicate the existence of nanosheets edges^16^, thus further confirms the lamellar structure of rGO. Fig. 1b shows the FT-IR spectra of GO and rGO. Apparently, the FTIR spectra were significantly different. By comparison, the carbon-carbon double bond at 1570 cm^−1^ and the carbon oxygen bond at 1205 cm^−1^ became stronger than GO, which suggests that the electronic conjugation is renovated after reduction, and carbon-oxygen-carbon structure became primary by replacing hydroxyl and carboxyl structure in GO^17^.

### Inactivation enhancement of *E*. *coli* in three-dimensional BDD-rGO system

**Figure 2:**
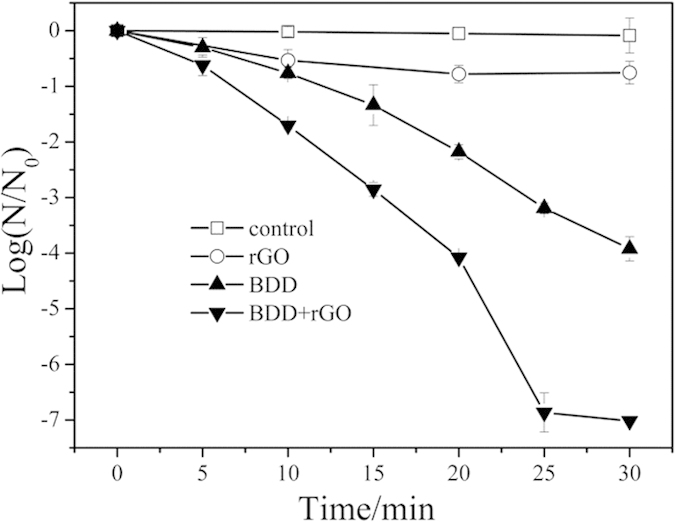
Inactivation of *E. coli* in different disinfection processes: (1) Control; (2) 1 μg ml^−1^ rGO; (3) BDD, 15 mA cm^−2^; (4) BDD with 1μg ml^−1^ rGO, 15 mA cm^−2^. Initial *E. coli* concentration: 10^7 ^CFU/ml, Na_2_SO_4_: 0.05 M.

The inactivation effect of *E. coli* was conducted under different disinfection conditions, i.e. only rGO, only BDD, BDD-rGO (Fig. 2). No obvious *E. coli* death observed without electricity at room temperature in 0.05 M Na_2_SO_4_ solution (see the control in Fig. 2), suggesting *E. coli* can keep a stable survive rate in the solution. When adding 1 μg ml^−1^ rGO but without electrolysis function in the same condition, *E. coli* was inactivated by about 0.7 log in 20 min, then kept unchanged even after several hours. On the other hand, the control experimental results (Fig. S3) indicated that the influence of hydrazine residual was almost negligible. *E. coli* demised strikingly in BDD electrochemical system with no rGO added, almost 4 log *E. coli* inactivated in 30 minutes. However, as high as 7 log *E. coli* was killed in BDD-rGO system with only 25 minutes (more outstanding performance was achieved under the optimized conditions, see Fig. S4), and no vital sign can be detected even after cultivation. That is, by adding rGO to construct a three-dimensional electrochemical system, the inactivation efficiency was improved more than 100%.

### Various influences on disinfection performances of BDD-rGO

To investigate the electrochemical ability of BDD-rGO system, influencing factors such as current density, electrolyte concentration, rGO concentration and reduction degree were systematically considered.

**Figure 3:**
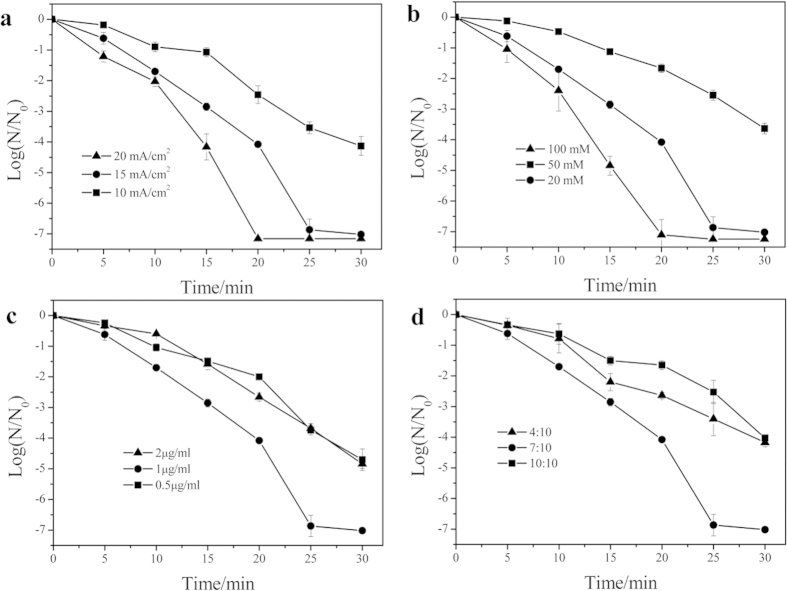
Various influences on disinfection performances of BDD-rGO: (**a**) Inactivation of *E. coli* in 0.05 M Na_2_SO_4_ electrolyte at different current density; (**b**) Inactivation of *E. coli* in different Na_2_SO_4_ electrolyte concentration at 15 mA cm^−2^ ; (**c**) Inactivation of *E. coli* in BDD electrochemical system with different rGO concentration; (**d**) Inactivation of *E. coli* in BDD electrochemical system with different rGO reduction degree. Initial *E. coli* concentration: 10^7^ CFU/ml.

The electrochemical experiment was conducted by varying current density of 10, 15 and 20 mA cm^−2^ respectively at given conditions. As shown in Fig. 3a, the inactivation efficiency increased gradually with increasing current density due to enhancement of hydroxyl radical generation^18^.

The effect of electrolyte concentration is presented in Fig. 3b. Increasing Na_2_SO_4_ concentration from 0.02 M to 0.1 M led to a continued increase of *E. coli* death rate. This implies that electro-generated S_2_O_8_^2−^ is expected to increase with increasing Na_2_SO_4_, which played an inevitable role in the electrochemical disinfection in addition to hydroxyl radicals^18^.

Figure 3c shows the effect of rGO concentration on *E. coli* inactivation. With increase of rGO concentration from 0.5 to 2 μg/ml, the highest antibacterial ability appeared at 1 μg/ml rGO. As rGO concentration increased from 0.5 to 1 μg/ml, the disinfection ability was augmented due to the increased synergic effect by rGO. However, once rGO is hydrophobic, it is much easier to aggregate, thus limiting the synergic effect in BDD-rGO system.

In order to investigate the effect of rGO reduction degree, different amount of reductant hydrazine was added to control the weight ration of hydrazine to GO as 4:10, 7:10, 10:10, respectively. A charaterization of different reduction degree of rGO by full-wave UV spectra was conducted and displayed in Figure S2a. After reduction, the absorption peak of GO gradually red shifted, and the higher the reduction degree is, the longer the wavelength is. Fig. 3d shows much higher disinfection ability in the system at rGO reduction degree of 7: 10.

It was found that higher rGO reduction degree would correspond to higher carbon ration, which was proportional to the conductivity in a electrochemical process. In the BDD-rGO system, rGO with higher reduction degree displayed higher electrical conductivity for promoting synergic interaction. However, the excessively higher reduction degree would result in the more sevious aggregation^17^, restricting the promotion effect of *E. coli* disinfection by rGO. After 5 days’ aggregation, rGO with the largest reduction degree, i.e. 10:10 (w/w) of hydrazine to GO, aggragated more seriously than others and displayed the lowest inactivation log of *E. coli* (see Fig S2b-c); while the 4:10 and 7:10 (w/w) of hydrazine to GO still kept perfect dispersibility, suggesting better disinfection performance.

To compare the influence on antibacterial ability by the above four factors, sensitivity analysis of *E. coli* fatality rate was made at the same disinfection time. Results displayed in the FigS5 suggest that current density concerns the most, followed by reduction degree, electrolyte concentration and rGO concentration.

### Synergic mechanism of BDD- rGO disinfection

**Figure 4:**
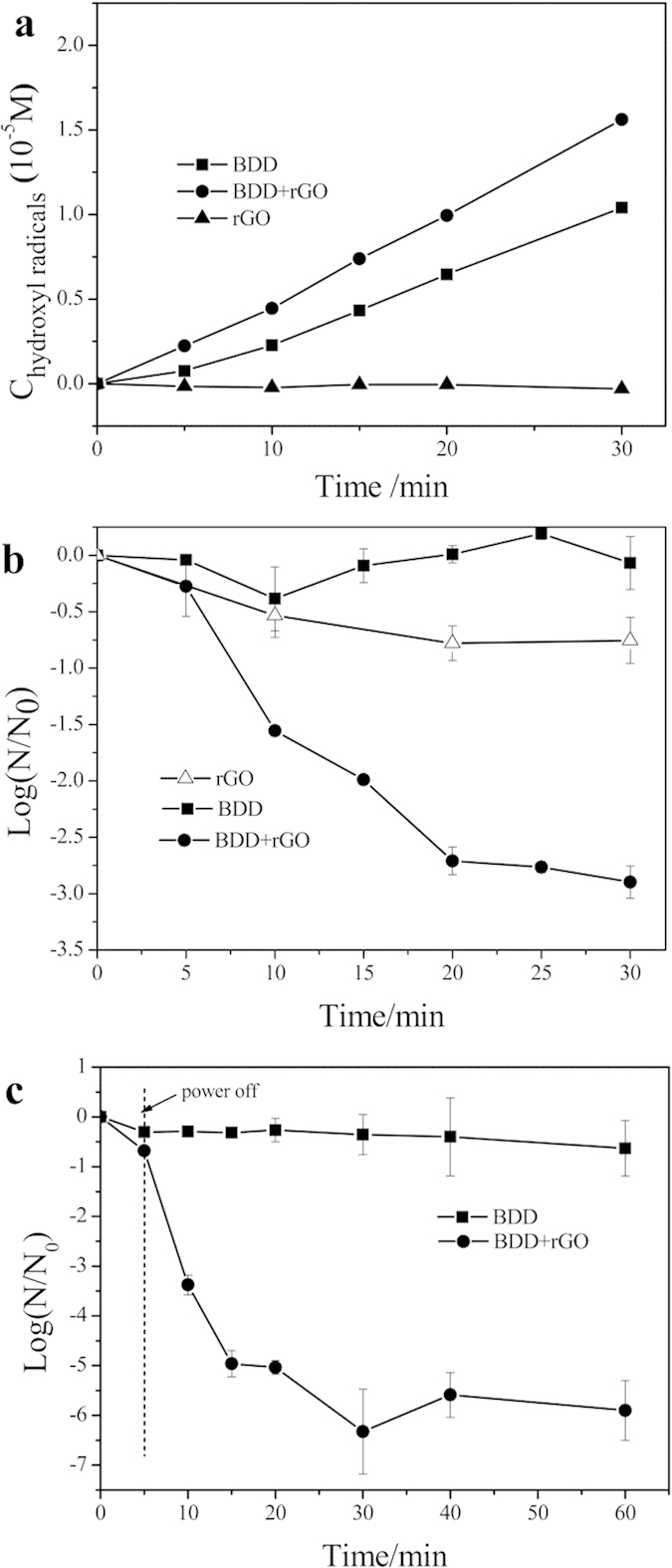
Synergic disinfection mechanism of BDD- rGO: (**a**) Concentration of hydroxyl radicals produced in different disinfection process; (**b**) Electric field effect towards rGO: inactivation of *E*. *coli* with excess methanol in process of rGO, BDD and BDD-rGO; (**c**) Capacitance effect of rGO: inactivation of *E. coli* in BDD and BDD-rGO, power cut off at 5 min. Initial concentration: 10^7^ CFU/ml, current intensity: 15 mA cm^−2^, Na_2_SO_4_: 0.05 M, rGO concentration: 1 μg ml^−1^.

Previous studies have proved that hydroxyl radicals played an important role in BDD electrochemical disinfection^5^. Similarly, hydroxyl radicals generation in rGO, BDD and BDD-rGO systems were detected respectively under the same conditions. In Fig. 4a, no production of hydroxyl radicals has been detected as rGO alone in Na_2_SO_4_ solution. Furthermore, BDD-rGO system produced 25% higher hydroxyl radicals than BDD. This implied that extra hydroxyl radicals were produced in the three dimensional electrochemical system. With presence of rGO in the BDD system, actual area of electrodes was apparently expanded, which would be of great help to generate more hydroxyl radicals; on the other hand, the delayed recombination of the electron–hole pairs would lead to more efficient catalysis production of hydroxyl radicals.

Special role of the BDD driven electric field on rGO disinfection was also investigated by adding excess methanol to eliminate the function of hydroxyl radicals, and the results were presented in Fig. 4b. In absence of hydroxyl radicals, BDD electrochemical system almost lost its disinfection ability, while BDD-rGO electrochemical system still had 3 log inactivation of *E. coli* after 30 minutes. Furthermore, rGO in presence of BDD driven electric field had much stronger antibacterial ability than rGO alone. Although the charge transfer between negative charged cell membrane and electronic acceptor was proposed as the dominant antibacterial mechanism, the p-type boron doped diamond electrode played as an electronic pump for driving the charge transfer and facilitating the electronic mobility between cell membrane and rGO.

Interestingly, when the power was cut off after 5 minutes’ electrolysis in BDD-rGO system, there was still excellent antibacterial performance in the following 15 minutes, i.e. even 4.5 log *E. coli* drastically inactivated (Fig. 4c), which could hardly be detected in a BDD system in absence of rGO. The sustained disinfection ability without power was benefited from the capacitance of rGO^19^. Although the log decrease was dramatically fast, it could not completely kill all the *E. coli* at initial concentration of 10^7^ CFU/ml.

## Discussions

A three-step rGO antimicrobial mechanism was proposed^20^ in the single system without power. Firstly, bacteria transferred to the surface of rGO nanosheet; then the cell membrane was ruptured by the sharp edges of nanosheets due to the direct contact; lastly, a charge transfer between the negatively charged membrane of *E. coli* and the electron acceptor (rGO)^21^,^22^ arose afterwards. Simultaneously, it has been proved that the microbe particles transporting to carbon nanotube surfaces can be increased by an external electric field^9^. Hence, it could be deduced that each of the rGO nanosheets would act as a tiny electrode in the three dimensional electrochemical oxidation systems, and thus greatly expanded the actual area of electrodes. As rGO was homogeneously dispersed in the solution containing *E. coli* cells, bacterial migration to rGO surface became faster with help of the BDD driven electro field, thus accelerated the disruption of cell membrane by the sharp edges of rGO. The disruption of *E. coli* can be enhanced through the expanded electro field. Moreover, quantum tunnel effect can result in an extremely high current-carrying efficiency of rGO, as gate voltage tuned the Fermi level across the charge neutrality point^23^. High current-carrying efficiency means the significant enhancement of charge transfer between bacterial membrane and rGO. Above all, stronger membrane stress was proposed in BDD-rGO electrochemical systems due to the expanded electro field and charge transfer.

**Figure 5:**
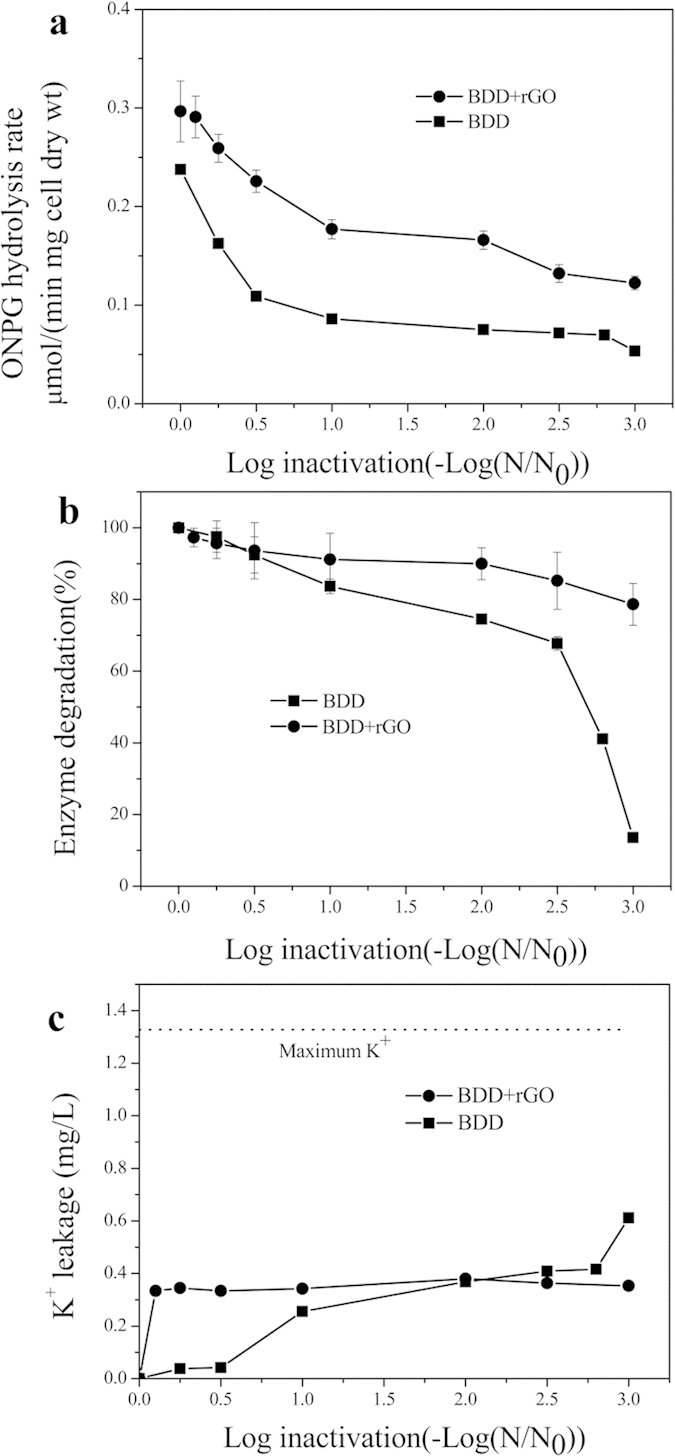
Membrane tests during the disinfection processes of BDD and BDD-rGO: (**a**) Cell permeability change as a function of *E. coli* inactivation assessed based on ONPG hydrolysis rate; (**b**) Degradation of intracellular β-D-galactosidase as a function of *E. coli* inactivation (pH 7.1, buffer condition); (**c**) K^+^ leakage of *E. coli* inactivation. Initial concentration: 10^8^ CFU/ml, current intensity: 15 mA cm^−2^, Na_2_SO_4_: 0.05 M.

The cell wall permeability can be indicated based on the rate of ONPG (o-nitrophenyl-β-D-galactopyranoside) hydrolysis. As a probe, ONPG can react with the intracellular enzyme of *E. coli*, i.e. β−D-galactosidase, to form a chromogenic substrate. The hydrolysis rate of ONPG is directly related to its ability of penetrating into *E. coli* cells and hence the damage of cell surface^24^. To evaluate the rupture degree of *E. coli* membrane during the disinfection, degradation kinetics of ΟΝPG was conducted and the results were shown in Fig. 5a. Control test confirmed that no measurable β−D-galactosidase was released to the reaction system during disinfection process. By comparison, BDD-rGO electrochemical system holds higher ONPG hydrolysis rate than that of BDD under the same inactivation level, indicating a heavier and faster cell membrane damage in BDD-rGO system.

To further explore how the cells were inactivated, the total amount of residual enzyme was analyzed by ONPG after all the cells were lysed through ultrasound. According to Fig. 5b, the enzyme degradation in BDD was faster than that of BDD-rGO under the same inactivation level, which suggested that the existence of residual cells in two systems were quite different even the number of killed *E. coli* was the same, i.e. there are more “damaged but existed” cells in BDD-rGO system.

The enzyme experiments confirmed that cell membrane damage was a major contributor to high increment of antibacterial ability in the BDD-rGO system. Negative charged bacterial migrated to the surface of rGO under the effect of electric field and then suffered a heavier membrane stress from both physical contact and charge transfer.

To confirm this phenomenon, the K^+^ leakage from *E*. *coli* cells during the disinfection process was also detected by FAAS and depicted in Fig. 5c. Apparently, in BDD-rGO system, K^+^ leakage raised very fast in the early stage of electrolysis, then stayed almost unchanged. While in BDD system, the K^+^ leakage slowly increased in the early stage of 1 log inactivation, then arrived at a platform, and continued to increase after 2.8 log inactivation. Cell membrane damaged quickly with gradual inactivation of *E. coli* cells in BDD-rGO electrochemical system. However, cell damage was accompanied with cell dispel in BDD electrochemical system, which exhibited a drastic release of intracellular contents including the damage of organelles only after 2.8 log inactivation.

**Figure 6:**
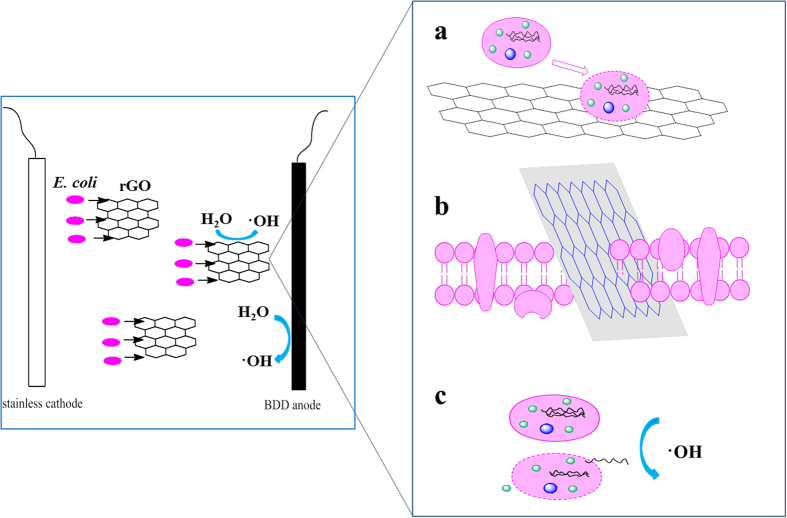
Schematic graphs to illustrate the proposed antibacterial mechanism in the BDD-rGO system. Left graph shows the promoted migration of *E. coli* to the surface of rGO. Right graph shows the three death mechanism of *E. coli*: (**a**) charge transfer between negative charged cell membrane and electronic acceptor rGO; (**b**) disruption of cell membrane by sharp edges of rGO nanosheets; (**c**) oxidation by·OH.

Quite different disinfection mechanism of *E. coli* cells in BDD electrochemical system and BDD-rGO system could be revealed. *E. coli* cells were killed faster in BDD-rGO system not only because of the production of extra hydroxyl radicals, but also the stronger membrane stress resulted from the acceleration of electric field to the permeable of cell membrane by rGO (Fig. 6).

**Figure 7:**
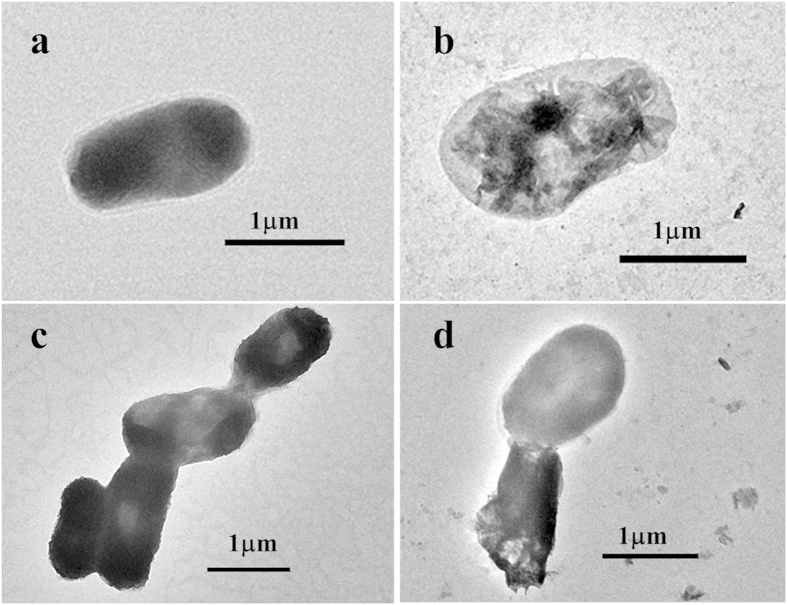
TEM images of *E. coli*: (**a**) Untreated cells; (**b**) Cells after 150 min with rGO; (c) Cells in BDD electrochemical system after 60 min electrolysis; (**d**) Cells in BDD-rGO electrochemical system after 30 min electrolysis.

Figure 7 shows the TEM images of *E. coli* morphology before and after treated by rGO, BDD electrochemical system and BDD-rGO electrochemical system. Obviously, untreated cells are round and full (Fig. 7a). After inactivation by rGO (Fig. 7b), some cells become apparent, which is correspond to ‘type B cells’ indicated by Tu^15^, of which graphene nanosheets extracted phosphollpids from the membrane. The *E. coli* cell treated by BDD electrolysis as shown in Fig. 7c, cells became long, thin and sticky, which proved a corrosive and intercellular damage of membrane^2^. While Fig. 7d displayed that rGO penetrated into the cell and caused vulnerable damage. These results confirmed the different disinfection mechanism among rGO, BDD electrochemical system and BDD-rGO electrochemical system from a visualized point of view.

## Methods

### Preparation and characterization of reduced graphene oxide(rGO) nanosheet dispersion

The reduced graphene oxide(rGO) nanosheet dispersion was synthesized strictly according to Li Dan^17^. The purified graphite oxide (GO) was first obtained through a modified Hummer’s method, followed by ultrasonication to purify and get GO dispersion. Afterwards, 100 ml GO dispersion with the concentration of 0.25 mg/ml, 35 μl hydrazine solution (50% w/w) and 375 ml ammonia solution (25% w/w) was mixed and kept in an oil bath with the constant temperature of 95 °C for 1 h. Lastly, the mixture was filtered by the cotton to get rGO nanosheet dispersion of ca. 200 μg/ml.

### Electrochemical disinfection experiments and antibacterial ability evaluation

Batch electrochemical experiments were carried out at the room temperature in a 400 ml beaker as the electrolytic cell. BDD electrode (CONDIAS GmbH, Germany) of 4 cm^2^ geometric area and a stainless steel electrode with the same area were separately served as anode and cathode, with the electrode gap of 1 cm. The power supply was provided by a DC power source under galvanostatic conditions.

As the indicator microorganism herein, *E. coli C3000* was firstly cultured in a LB (Luria-Bertani) medium at 37 °C for 13 h. The cells were then obtained through centrifugation at 6000 rpm for 8 minutes, followed by washing to remove residual nutrient ingredient. Before each disinfection experiment, the *E. coli* solution with initial concentration of ca. 10^7^ CFU/ml was suspended in electrolytes and stirred at least for 20 min.

Throughout a typical disinfection process, a 250 ml electrolytic solution containing *E. coli* suspension was continuously stirred by a magnetic stirring apparatus. Samples were withdrawn at different time intervals to determine the concentration of survival microorganisms. Once the samples were taken, excess sodium thiosulfate solution of 10 mM was immediately added to capture residual •OH in order to terminate the oxidation reaction. The number of viable cells after disinfection was determined by plating and counting colonies after 13–16 h incubation in a nutrient agar media at 37 °C. The survival data was expressed as log N/N_0_, where N_0_ is the number of initial colonies, and N is the number of colonies after electrochemical disinfection.

### Detection of hydroxyl radicals

Hydroxyl radicals were detected through the reaction with N,N-dimethy-p-nitrosoaniline (RNO) according to Comninellis^25^, as displayed by Equation (1)-(2). The experiment was conducted in the same reaction system with constant pH 7.1 controlled by phosphate buffer, and current density of 15 mA cm^−2^, containing 3 × 10^−5^ M RNO. RNO was a kind of spin traps, which has a large reaction rate of 1.2 × 10^10^ M^−1^ s^−1^ and high selectivity to·OH. By measuring the yellow matter (RNO) at 440 nm using a UV-vis spectrophotometer (UV-1800, Shimadzu), the production of hydroxyl radicals can be determined through the reaction proportion to RNO.


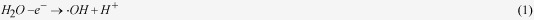



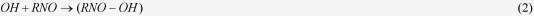


### Characterization of cell Membrane

ONPG (o-nitrophenyl-β-D-galactopyranoside) experiments were conducted to detect the damage level of cell membrane according to Cho^25^. *E. coli* was employed by an induced cultivation to enhance the synthesis of β-D-galactosidase. After disinfection, *E. coli* sample was mixed with 5 mM ONPG in phosphate buffer solution (PBS). The solution was sampled at different time intervals and immediately mixed with 1 M sodium carbonate/bicarbonate buffer at pH 10 to stop the reaction. The yellow reaction product, i.e. o-nitrophenol, was measured at 420 nm by the UV-vis spectrophotometer to determine hydrolysis kinetics. The degradation of enzyme inside *E. coli* was analyzed after ultrasonication (Ningboxinzhi Biotechnology Ltd., China), by which the intracellular enzyme was released to the suspension. Then ONPG hydrolysis assay was conducted as described above. K^+^ leakage was measured by Flame Atomic Absorption Spectrometer (FAAS, Analytik Jena AG, Japan). Sample were collected after disinfection and treated by filter membrane (0.22 μm).

The Transmission electron microscope (TEM) analysis was conducted by Tecnai G2 T20 to describe the morphology of *E. Coli* cell. Sample suspension after disinfection was centrifuged and washed to remove the residual salt. Then it was re-suspended in distilled water and drop onto the cupper grids for TEM observation.

### Sensitivity analysis on disinfection performances of BDD-rGO

According to Seshan *et al*^26^, sensitivity of *E. coli* fatality rate is defined as equation (3) which can be used for evaluation of sensitivity subject to influences of the four factors at the same disinfection time.


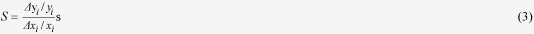


As results, the confidence level is found greater than 95%.

## Additional Information

**How to cite this article**: Qi, X. *et al.* Synergetic antibacterial activity of reduced graphene oxide and boron doped diamond anode in three dimensional electrochemical oxidation system. *Sci. Rep.* **5**, 10388; doi: 10.1038/srep10388 (2015).

## Supplementary Material

Supplementary Information
