# Supplementary material for: Synergetic antibacterial activity of reduced graphene oxide and boron doped diamond anode in three dimensional electrochemical oxidation system
Source: Sci Rep. 2015 May 21;5:10388. doi: 10.1038/srep10388 (PMC4440036; doi:10.1038/srep10388)
Supplement: Supplementary Information [file srep10388-s1.doc]

**Supplementary Information**

**Synergetic antibacterial activity of reduced graphene oxide and boron doped diamond anode in three dimensional electrochemical oxidation system**

Xiujuan Qi ^a^, Ting Wang ^b^, Yujiao Long ^b^, Jinren Ni ^a, b, *^

*^a^ School of Environment and Energy, Peking University Shenzhen Graduate School, Shenzhen 518055, China*

*^b^ College of Environmental Science and Engineering, Peking University; Key Laboratory of Water and Sediment Sciences, Ministry of Education, Beijing 100871, China*

* Corresponding author: Tel: +86-10-62751185; Fax: +86-10-62756526.

E-mail address: nijinren@iee.pku.edu.cn (J.R. NI)

**1. Supplementary figures**


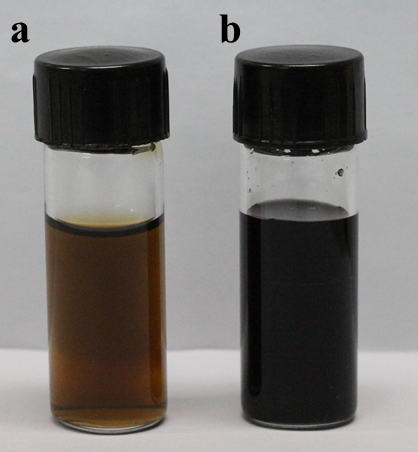


**Figure S1.** Typical color of (a) GO and (b) rGO.


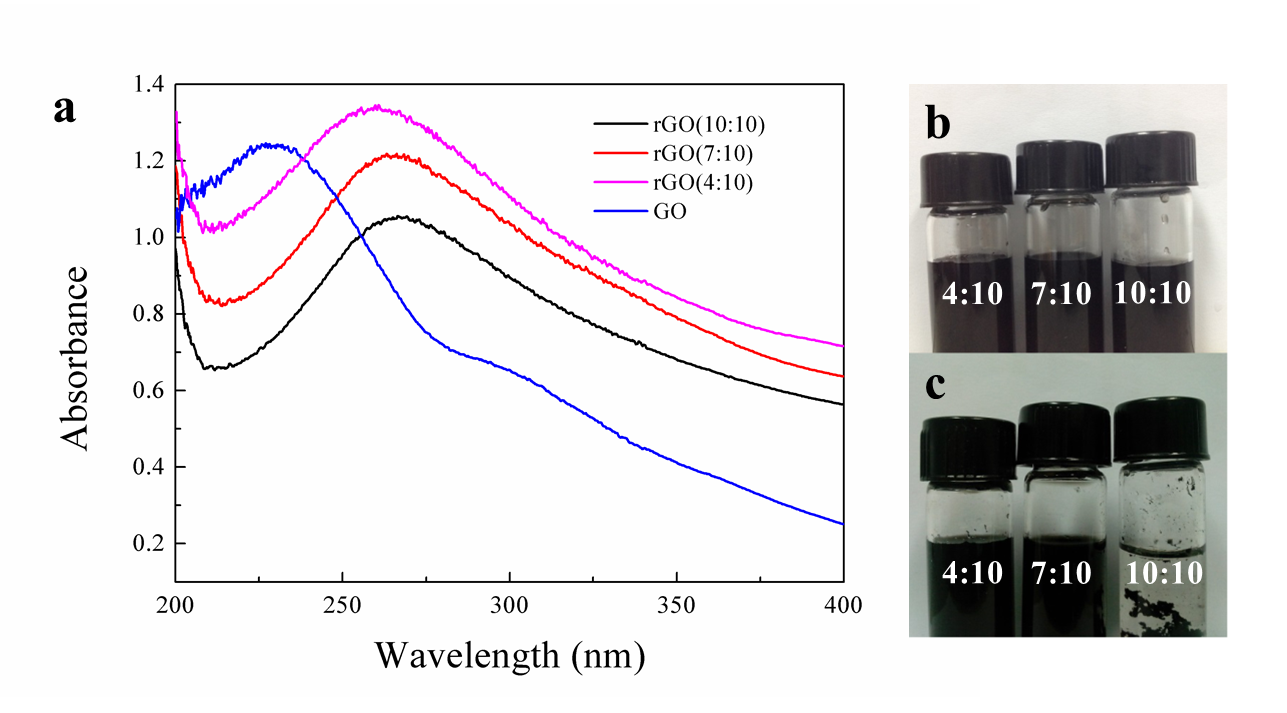


**Figure S2**. (a) Full-wave UV spectra of GO and different reduction degree of rGO; (b) Photograph of rGO (with different reduction degree) immediately after preparation. (c) Photograph of rGO (with different reduction degree) 5 days after preparation.

**2. Tests on influence of hydrazine residual**

To identify the influence of hydrazine on the death of *E. coli* in our disinfection system (rGO, BDD, BDD+rGO), control experiment with hydrazine concentration of 1.75×10^-3^ μg ml^-1^ (determined under the hypothesis of no reaction of hydrozine with rGO during redution process) is conducted, and the experimental results are given in the following table and photo:

| **Table S1｜Survival rate of *E. coli* in hydrazine and rGO solution at different time interval** | | | |
| --- | --- | --- | --- |
|  | 0 min | 10 min | 30 min |
| Hydrazine | 1 | 1.04 | 0.95 |
| rGO | 1 | 0.89 | 0.83 |
| Note: Initial E. *coli* concentration: 10^7^ CFU/ml; Na_2_SO_4_: 0.05M; Hydrazine: 1.75×10^-3^ μg ml^-1^; rGO: 1μg ml^-1^. | | | |


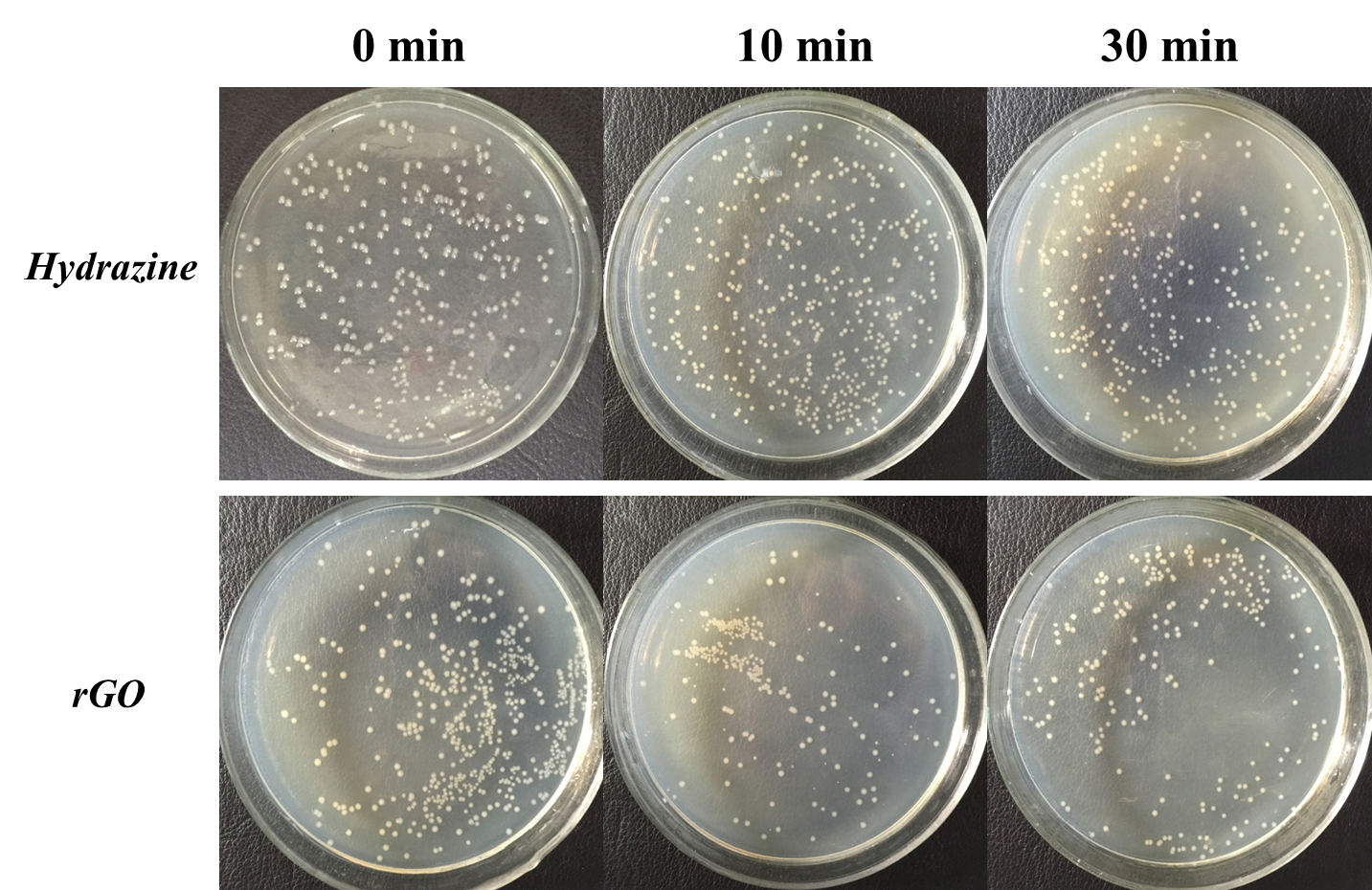


**Figure S3.** Photographs of survival *E. coli* colonies on agar culture plates (10^5^ times dilution) at different time interval (0min, 10min, 30min), with the initial *E. coli* concentration: 10^7^ CFU/ml.

The experimental results show that the influence of hydrazine on the *E. coli* death is almost negligible in our disinfection system.

**3. BDD and BDD-rGO disinfection processes in all optimized conditions**

BDD-rGO system performs excellently by killing *E. coli* within only several minutes, which can be further demonstrated by comparison of the performance of BDD and BDD-rGO system under the same conditions.

| **Table S2｜Survival number of *E. coli* in BDD and BDD-rGO disinfection processes in all optimized conditions at different time interval** | | | |
| --- | --- | --- | --- |
|  | 0min | 5min | 8min |
| BDD | 4.82×10^7^ | 3230 | 350 |
| BDD+rGO | 5.05×10^7^ | 1330 | 0 |
| Note: Initial *E. coli* concentration: 10^7^ CFU/ml; Na_2_SO_4_: 0.1M; current density: 20 mA cm^-2^; reduction degree : 7:10; rGO: 1μg ml^-1^. | | | |


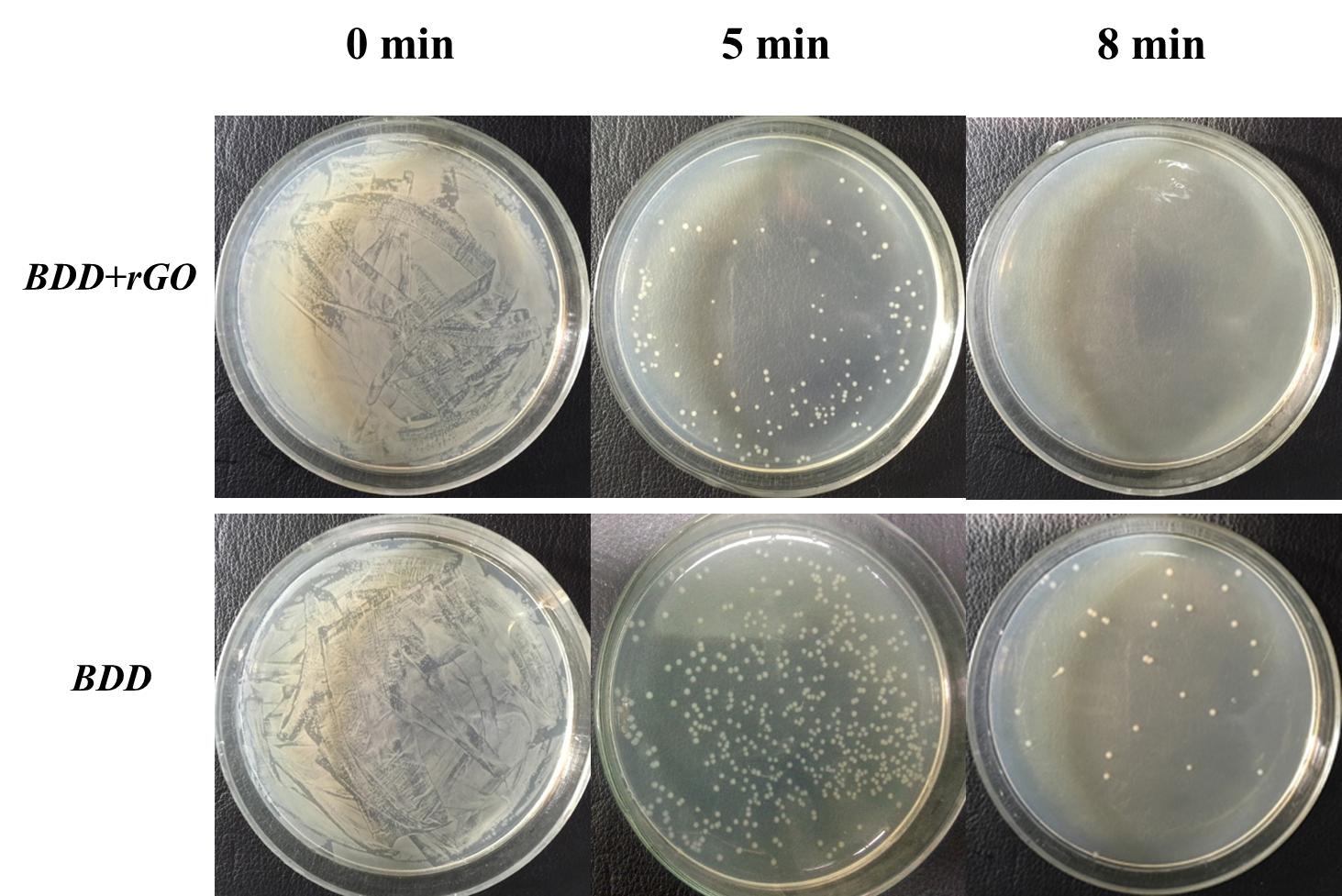


**Figure S4.** Photographs of survival *E. coli* colonies on agar culture plates (10 times dilution) under different disinfection processes (BDD with 1μg ml^-1^ rGO; BDD) in all optimized conditions (20 mA cm^-2^, 0.1M Na_2_SO_4_, 1 μg ml^-1^ rGO, reduction degree of 7:10) at different time interval (0min, 5min, 8min), with the initial *E*. *coli* concentration: 10^7^ CFU/ml.

**4. Sensitivity analysis**

The influence on antibacterial ability of BDD-rGO by four variables (current density, electrolyte concentration, rGO concentration and reduction degree of rGO) was compared through sensitivity analysis of *E. coli* fatality rate at the same disinfection time. Results displayed in the following figure suggest that current density concerns the most, followed by reduction degree, electrolyte concentration and rGO concentration.


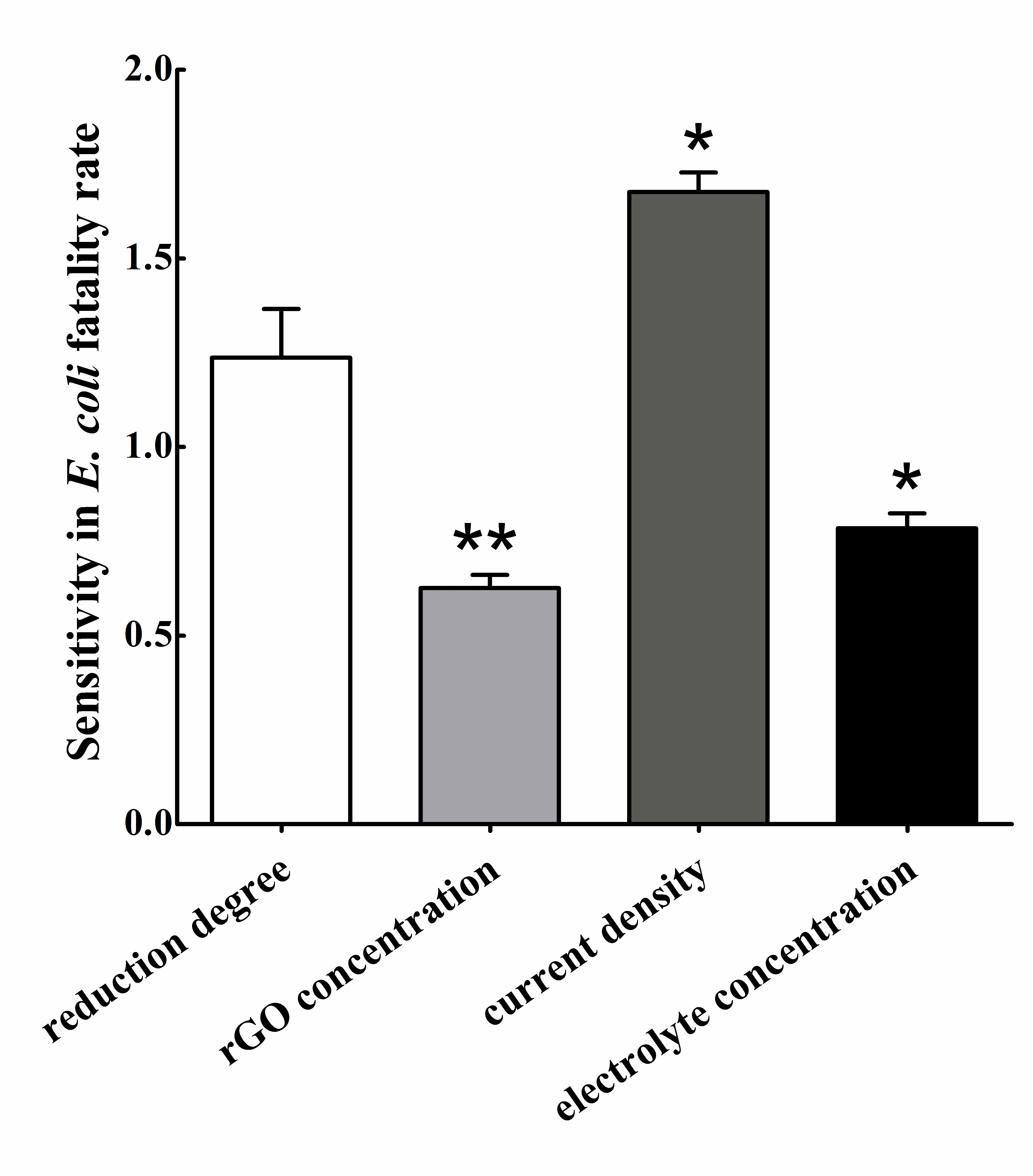


**Figure S5.** Variation of *E. coli* fatality rate to variables of current density, electrolyte concentration, rGO concentration and reduction degree of rGO, respectively. *p < 0.05, **p < 0.01 (compared with reduction degree, one-way ANOVA with Bonferroni’s multiple-comparisons test, n=3).
